# Reduced Growth and Nitrogen Uptake During Waterlogging at Tillering Permanently Affect Yield Components in Late Sown Oats

**DOI:** 10.3389/fpls.2019.01087

**Published:** 2019-09-12

**Authors:** Iduna Arduini, Marco Baldanzi, Silvia Pampana

**Affiliations:** Department of Agriculture, Food and Environment, University of Pisa, Pisa, Italy

**Keywords:** *Avena byzantina*, *Avena sativa*, grain-yield-components, N-status, recovery, roots, tillers, waterlogging

## Abstract

In Mediterranean Europe, winter cereals can experience soil waterlogging starting from crop establishment up to stem elongation and, in late sowings, this stress is combined with temperatures favorable to plant metabolism. Oats response to waterlogging has been rarely investigated, but these species seems to recover better than other cereals. In a 2-year experiment, *Avena sativa* and *Avena byzantina* were sown at the end of winter in pots placed outdoors. At the two-tiller stage, plants were exposed to waterlogging for periods ranging from 0 to 35 days. The dry weight and the N-concentration of shoots and roots were determined on waterlogged plants and drained controls at the start and the end of each waterlogging period, and at maturity. At maturity, the grain yield and its components were determined. To relate oat response to its specific morphological and developmental traits, results were compared to the published results in wheat and barley. Both oat species suffered severe damage during waterlogging: the uptake of nitrogen and the N-concentration of shoots were reduced after 7 days, tiller initiation and root growth after 14 days, and shoot growth after 21 days. All plants survived waterlogging, and the relative growth rates of roots and shoots and the net uptake rate of nitrogen were resumed during recovery. Nevertheless, at maturity, the straw and root biomass were markedly lower with all waterlogging durations, and grain yield decreased by 42% up to approximately 81% following an asymptotic equation. The most affected yield components were the number of panicles per plant and the number of kernels per panicle, but their relative sensitivity changed according to waterlogging duration. The slight increase in tiller fertility in response to short waterlogging and the small and irregular decrease in the number of kernels per spikelet suggest that the two oats could recover the initiation and size of inflorescences better than other winter cereals. Despite this, waterlogging in spring was highly detrimental to these oats because of severe damage under waterlogging and because of the inability to initiate new tillers and adequately resume root growth during recovery, once plants had achieved the phase of stem elongation.

## Introduction

Soil is considered waterlogged when excess water saturates the soil pores, with either no layer or a very fine layer of water on the soil surface, so that the gas exchange of roots with the atmosphere is inhibited (Sasidharan et al., 2017). Waterlogging is a major abiotic constraint on the growth and development of agricultural crops and occurs in many regions worldwide because of poor drainage and/or excessive rainfall. Crop losses due to waterlogging are expected to increase as a consequence of increased extreme precipitation events associated with climate change (Herzog et al., 2016).

Waterlogging is a compound stress that severely changes the soil environment, first by reducing oxygen availability. It is commonly accepted that anaerobiosis takes place within a few hours after the soil has been saturated and that the rate of depletion is greatly affected by soil depth and temperature (Belford et al., 1985; Cannell et al., 1985). Additional negative conditions caused by waterlogging in soil are the accumulation of CO2, ethylene, and biochemically reduced compounds in the root environment, generally combined with nutrient deficiency (Pang et al., 2007; Herzog et al., 2016). In particular, lower soil nitrogen has been reported as a consequence of denitrification and N leaching (Bronson and Fillery, 1998; Nguyen et al., 2018).

High oxygen-consuming tissues, such as actively dividing root meristems and those involved in mineral uptake, are the first targets of depleted oxygen, and their cells enter an “energy crisis” within a short time (Setter and Waters, 2003; Loreti et al., 2016). Brisson et al. (2002) observed the first effects of waterlogging on the growth of wheat roots after 48 h. Plants cope differently with occasional waterlogging toward which they display a variety of tolerance mechanisms such as the formation of aerenchyma in roots and the down-regulation of energy requirements (Herzog et al., 2016; Ploschuk et al., 2018).

Crop tolerance to waterlogging has been variously defined in the literature. Physiological tolerance takes into account the survival or the maintenance of growth rates similar to drained conditions under waterlogging, whereas agronomic tolerance is based on the maintenance of relatively high grain yields, despite waterlogging exposure during the growth cycle (Setter and Waters, 2003). Thus, the agronomic tolerance takes into account total plant behavior during and after the stress and is, therefore, the result of the direct effects of waterlogging on plant growth and the recovery ability after the waterlogging ceases.

Recovery upon drainage is particularly important for winter cereals grown in Mediterranean regions, where rainfall concentrates in the period November–April and drought can occur during the late reproductive phase. In these conditions, rapid growth of deep roots following drainage is required to obtain sufficient water to flower effectively and to complete seed ripening (Colmer and Greenway, 2011). In this area, winter cereals are traditionally sown from autumn to the end of winter and are, therefore, likely to experience waterlogging at different growth stages. In dependence on sowing time, the stress can be combined with either winter or spring temperatures, which can affect both the severity of damages and the time to recover. The sensitivity to waterlogging stress and the subsequent effects on grain yield is reported to depend on the development stage during waterlogging, on the duration of the event, and on the external conditions, primarily soil parameters and temperature (Setter and Waters, 2003; Arduini et al., 2016a; Ploschuk et al., 2018). Plant sensitivity to waterlogging was reported to be higher with higher temperatures, because of faster oxygen depletion from the soil (Belford et al., 1985; Setter and Waters, 2003) and because higher temperatures enhance transpiration and favor plant metabolism, thus increasing both energy consumption (de San Celedonio et al., 2014; Herzog et al., 2016; Loreti et al., 2016) and damages to growing points (Malik et al., 2002). According to de San Celedonio et al. (2014), losses in wheat and barley yield were higher with delayed sowings and when waterlogging occurred around anthesis compared to tillering, whereas according to Ghobadi and Ghobadi (2010), the susceptibility of wheat decreased with plant development from the first-leaf stage until stem elongation.

Most studies on waterlogging have focused on wheat and barley, while oat species have rarely been investigated (Mustroph, 2018). Nevertheless, oats rank sixth in global cereal production statistics, and in addition of being an important component of livestock feed, their cultivation has increased due to the demand for cosmetic uses and human nutrition, which is driven by their nutraceutical properties (Mahadevan et al., 2016; Finnan and Spink, 2017). In wheat and barley, damages caused by soil waterlogging include chlorosis and premature leaf senescence, reduced root growth, tillering, dry matter accumulation, number and weight of kernels, and increased floral sterility (Marti et al., 2015; Masoni et al., 2016; Arduini et al., 2016a; de San Celedonio et al., 2018; Ploschuk et al., 2018; Sundgren et al., 2018). Research of Watson et al. (1976) and Cannell et al. (1985) has suggested that oats recover better than other winter cereals from waterlogging stress, maybe because of the higher maintenance of green leaves during waterlogging and the higher tiller fertility at maturity (Setter and Waters, 2003).

Vegetative traits are similar in the three cereals, but differences are reported in the initial growth rate, which is slower in oats compared to wheat and especially to barley, and in the longer persistence of seminal roots in oats (Bonnett, 1961). The three species are highly responsive to nitrogen, and, in all of them, N supply during recovery was found to compensate, though only partially, for the waterlogging damage (Watson et al., 1976; Robertson et al., 2009; Rasaei et al., 2012). Conversely, oats differ from wheat and barley in the architecture of the inflorescence, in the timing of yield component determination and in the plasticity of them (Bonnett, 1966; Mahadevan et al., 2016).

The oat inflorescence is a panicle consisting of several branches grouped in clusters at the nodes of the main axis (Bonnett, 1966). Due to this morphology, oats have the potential to produce other spikelets after the initiation of a terminal spikelet at the end of each branch and, thus, after the start of stem elongation (Sonego et al., 2000; Browne et al., 2006). In contrast, the inflorescences of wheat and barley are spikes, in which spikelets are initiated only at the end of the main axis. Their final number is largely determined at the start of stem elongation, either because of the formation of a terminal spikelet (wheat) or because later initiated primordia will abort (barley) (Arduini et al., 2010). In addition, oats have the ability to either abort or fill additional kernels per spikelet in response to assimilate availability later in the crop cycle compared to the other cereals (Finnan and Spink, 2017). All above differences delay to approximately the start of grain filling the determination of the final number of kernels per plant in oats, whereas this character has been definitively fixed earlier in the other cereals: around anthesis in barley and at early post-flowering in wheat (Finnan and Spink, 2017). In all cereals, kernel number per plant is a crucial yield determinant, as it is the yield component which is most strongly related to grain yield and also more variable in response to environmental and stress conditions (Mahadevan et al., 2016). It is a complex trait resulting from the number of fertile tillers per plant and the number of kernels per inflorescence, which in turn results from the product of the number of spikelets and the number of kernels per spikelet. According to Mahadevan et al. (2016), in oats, the variations of the number of kernels per plant in response to stress conditions were primarily driven by variations of the number of kernels per panicle, whereas in wheat by the number of spikes per plant.

Starting from this, we hypothesized that the better ability of oats to recover from waterlogging imposed at tillering compared to wheat and barley (Watson et al., 1976; Cannell et al., 1985; Setter and Waters, 2003) could largely rely on the later determination and higher plasticity of its inflorescence, which allowed plants to adjust panicle components to compensate for the lower tillering and spikelet initiation during the waterlogging stress.

For the present research, we choose *Avena sativa* L. (common oat) and *Avena byzantina* C. Koch (red oat) that are the two most cultivated oat species in Italy. The two crops cover approximately equivalent surfaces, the former in northern regions and the latter in southern regions. Both crops are traditionally sown in autumn, but high autumn rainfall often prevents sowing at optimal times, so spring sowings are also frequent. To the best of our knowledge, no research has been conducted on the waterlogging tolerance of *A. byzantina*. However, because of the different geographic distribution of the two crops, it is to be expected that it could be less tolerant to waterlogging than *A. sativa*.

Aiming to fill the lack of knowledge about the response of oats to waterlogging, and to give insight into both the direct effects of waterlogging on plant growth parameters and the ability to resume them during recovery, we exposed plants of *A. sativa* and *A. byzantina* sown at the end of winter to different waterlogging durations. In specific, our research aimed to: (i) assess the immediate damage of waterlogging on root and shoot growth, and on nitrogen uptake of the two oat species; (ii) evaluate their ability to recover biomass production and N-uptake from the end of waterlogging up to maturity; and (iii) assess the delayed effects of waterlogging on the vegetative biomass and the grain yield and its components, at maturity. To give a better understanding of oats response to waterlogging and to relate this to the oat-specific plasticity of yield components, our results were discussed in comparison to published results on wheat and barley.

## Materials and Methods

### Experimental Location and Weather Conditions

The research was carried out in 2015 (11 February–2 July) and 2016 (8 February–4 July) at the Research Centre of the Department of Agriculture, Food and Environment of the University of Pisa, Italy, which is located approximately 5 km from the sea (43° 40′ N, 10° 19′ E) and 1 m above sea level. The climate of the area is hot-summer Mediterranean (Csa) with mean annual maximum and minimum daily air temperatures of 20.2 and 9.5°C respectively, and a mean rainfall of 971 mm per year. Daily air minimum and maximum temperatures and rainfall were recorded throughout the entire period of the research by an automatic meteorological station located close to the experimental site.

**Figure 1:**
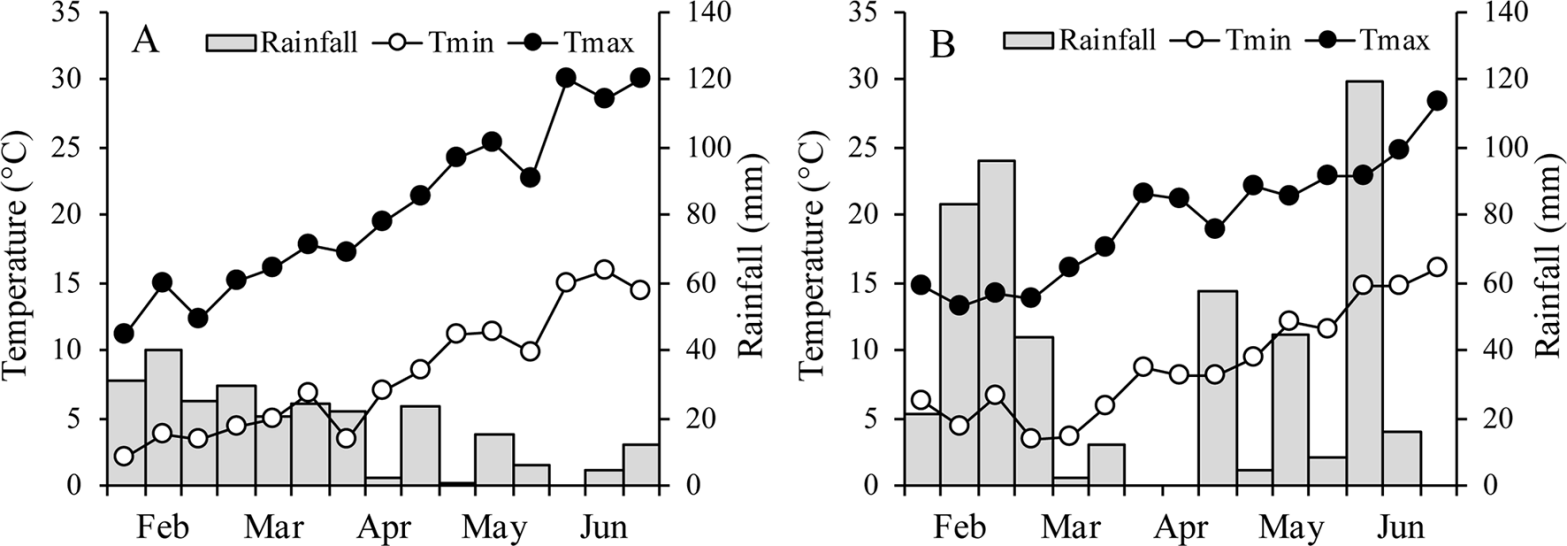
Air minimum and maximum temperatures and rainfall over the growing season of the two oats in 2015 **(A)** and 2016 **(B)**.

Between the two growth seasons, no differences in mean temperature were recorded during the entire vegetative phase of oats (14.3 and 14.4°C in 2015 and 2016, respectively), while the mean temperature during the waterlogging treatment was 18.6°C in 2015 and 20.0°C in 2016 (**Figure 1**). During the reproductive phase, however, the mean temperature was higher in 2015 (19.9°C) than in 2016 (18.4°C). In 2015, minimum temperatures were lower and maximum temperatures higher than in 2016, but the range was lower than 1°C. Rainfall varied considerably between years, with the growing season wetter in 2016 (509 mm) than in 2015 (only 257 mm).

### Experimental Design, Equipment, and Crop Management

The experimental design consisted of two oat species that were exposed to six waterlogging durations (0, 7, 14, 21, 28, and 35 days) at the tillering stage. We used the commercial cultivars Genziana for *A. sativa* L. (common oat) and Argentina for *A. byzantina* C. Koch (red oat), which are standard cultivars in Europe (Murariu et al., 2013). Both have medium-early growth cycles, medium-height, and a good resistance to lodging. In previous experiments carried out on wheat, durum wheat, and barley sown in autumn, we found that plants showed grain yield reductions when exposed to waterlogging at the tillering stage for more than 16 days (barley) and 20 days (wheat and durum wheat) (Masoni et al., 2016; Pampana et al., 2016; Arduini et al., 2016a). In the present study, we expected that the higher temperatures during waterlogging, imposed by the later sowing, would increase plant sensitivity at tillering. For this reason, we chose 35 days as the longest exposure time.

Plants were grown in 16-L pots made from polyvinyl chloride (PVC) tubes (80 cm long and 16 cm in diameter) fitted with a PVC base. A 30-mm diameter hole was drilled in the bottom of each pot, which was fitted with a 0.9-mm mesh to contain roots and substrate loss. In both years, pots were filled with a sandy-loam soil collected from a field previously cultivated with rapeseed. Differences in soil properties for the 2 years were negligible, and the average soil properties were 54.7% sand (2–0.05 mm), 33.4% silt (0.05–0.002 mm), 11.9% clay (< 0.002 mm), 7.6 pH, 0.7 g kg^−1^ total nitrogen (Kjeldahl method), 4.6 mg kg^−1^ available P (Olsen method), and 68.6 mg kg^−1^ available K (BaCl2-TEA method).

Oat species were sown on 11 February in 2015 and 8 February in 2016 (**Table 1**), which correspond to early spring sowings in the Mediterranean conditions. After emergence, the seedlings were thinned to five plants per pot, to mimic a field density of 250 plants m^−2^. Phosphorus and potassium were applied pre-planting as triple mineral phosphate and potassium sulfate, at the rates of 150 kg ha^−1^ of P2O5 and K2O. At sowing, all pots also received 30 kg N ha^−1^, as ammonium sulfate. As nitrogen application was found to reduce the detrimental effects of waterlogging on grain yield, in experiment 2, an additional 120 kg N ha^−1^ was applied as urea at the start of the recovery period.

**Table 1:** Timing of principal growth stages of *A. sativa* and *A. byzantina* in the two growing seasons.

Growth stage	BBCH Code	Growing Season	Species	
			*Avena sativa*	*Avena byzantina*
Sowing	00	2015	11 February	11 February
		2016	8 February	8 February

Emergence	09	2015	25 February	25 February
		2016	22 February	22 February
				
Beginning of tillering	21	2015 2016	15 March 21 March	13 March 18 March

Two tillers detectable	22	2015 2016	20 March 24 March	19 March 23 March

First node detectable	31	2015 2016	3 April 6 April	4 April 7 April

Full flowering	65	2015	7 May	11 May
		2016	11 May	13 May

Maturity	89	2015	2 July	2 July
		2016	4 July	4 July

During the entire growth cycle, the timings of the principal growth stages were recorded following the BBCH scale for cereals (Meier, 2001); weed control was conducted by hand hoeing, and weekly checks were conducted for the occurrence of diseases.

In both years, we filled and sowed 68 pots per species. Pots were placed outdoors and kept under drained conditions until plants reached the two-tiller stage (20 March 2015 and 24 March 2016). At these dates, four pots were harvested (T0), 24 pots were maintained in drained conditions, and 40 pots were exposed to waterlogging by placing them into containers (2 m x 1 m x 1 m) filled with water. A layer of 1 cm of free water was maintained above the soil surface throughout the period of waterlogging, to ensure that the soil was completely saturated by water. Accordingly, the treatment consisted of a stagnant soil waterlogging (Sasidharan et al., 2017).

To assess the extent to which *A. sativa* and *A. byzantina* are able to both resist and recover from waterlogging stress, the 68 pots per species were used for two combined experiments that were conducted in parallel.

### Experiment 1

Experiment 1 was aimed at assessing the effects of waterlogging on the growth and N uptake of plants harvested immediately after the end of exposure. Each year, the experimental design consisted of two species (*A. sativa* and *A. byzantina*), two growth conditions (drained control—C and waterlogged—WL) and six waterlogging durations (T0, T7, T14, T21, T28, T35). Four replicate pots were used for all combinations of treatments.

For this experiment, each year, 44 pots per species were harvested as follows: four pots before the WL treatment was imposed (T0) and, then, four pots kept under water (WL) and four drained pots (C) at week intervals (T7, T14, T21, T28, T35).

### Experiment 2

Experiment 2 was aimed at assessing if damage caused by the waterlogging imposed at tillering affected oat performance at maturity. Each year, the experimental design consisted of two species (*A. sativa* and *A. byzantina*) and six waterlogging durations (T0, T7, T14, T21, T28, T35). Four replicate pots were used for all combinations of treatments.

For this experiment, four pots per species were moved from the container filled with water to drained conditions at week intervals starting 1 week after waterlogging was imposed (T7, T14, T21, T28, T35). Then, pots were supplied with 120 kg N ha^−1^ and kept in drained conditions until plants reached maturity. For each species, T0 consisted of four pots that were kept in well-drained conditions throughout the entire growth cycle. These pots received N at the same time of the WL pots that were drained at T7. All pots, 24 per species at a whole, were harvested at maturity.

### Recovery From Waterlogging

The ability of the oat plants to recover from waterlogging stress was assessed by determining growth and nitrogen uptake between the end of waterlogging and maturity.

For each combination of treatments, the recovery of growth parameters and N uptake was calculated as the difference of the values recorded at maturity in experiment 2 and those of the correspondent combination of treatments recorded at the end of waterlogging in experiment 1.

### Plant Measurements

At all harvests, plants were manually cut at ground level, and roots were separated from the soil by gently washing to minimize loss or damage. In experiment 1, the number of culms per plant was recorded. In experiment 2, shoots were partitioned into culms+leaves, chaff, and grain. The number of culms and panicles per plant, and the number of spikelets per panicle were recorded. The mean kernel weight was determined, and the number of kernels per plant and kernels per spikelet were calculated. The harvest index was calculated as the ratio between grain yield and total aboveground biomass.

For the dry weight determination of roots and aerial parts, the samples were oven dried at 65°C to a constant weight. All plant parts were analyzed for nitrogen concentration using the micro-Kjeldahl standard method (AOAC, Association of Official Analytical Chemists, 2005). Nitrogen content was obtained by multiplying N concentrations by dry matter.

To determine the effect of waterlogging on the growth and nitrogen uptake of oats during and after waterlogging, the following indexes were calculated for each waterlogging duration and for the periods of recovery.

The absolute growth rates (AGR) of shoots and roots was calculated following Hunt (1990) as:

$$\mathrm{AGR}=\frac{W_{2}-W_{1}}{t_{2}-t_{1}}$$

where W is the dry biomass at the beginning (W_1_) and at the end (W_2_) of each period, and t_2_–t_1_ is the duration of the period. In experiment 1, t_1_ and t_2_ are two consecutive harvests, whereas in experiment 2, t_1_ corresponds to the end of each WL duration and t_2_ to maturity.

The relative growth rates (RGR) of shoots and roots was calculated following Hunt (1990) as:

$$\mathrm{RGR}=\frac{\ln W_{2}-\ln W_{1}}{t_{2}-t_{1}}$$

where W is the dry biomass of shoots or roots at the beginning (W_1_) and at the end (W_2_) of each period, and t_2_–t_1_ is the duration of the period. As for AGR, in experiment 1, t_1_ and t_2_ are two consecutive harvests, whereas in experiment 2, t_1_ corresponds to the end of each WL duration and t_2_ to maturity.

The net uptake rates (NUR) of nitrogen were calculated following Engels (1993) as:

$$\mathrm{NUR}=\frac{N_{2}-N_{1}}{t_{2}-t_{1}}\times \frac{\ln\left(R_{2}/R_{1}\right)}{R_{2}-R_{1}}$$

where N is the nitrogen content of the entire plant, R is the dry weight of the roots at the beginning (1) and at the end (2) of each period, and t_2_–t_1_ is the duration of the period. As for AGR, in experiment 1, t_1_ and t_2_ are two consecutive harvests, whereas in experiment 2, t_1_ corresponds to the end of each WL duration and t_2_ to maturity.

### Statistical Analyses

All results were subjected to analysis of variance separately for *A. sativa* and *A. byzantina*. To evaluate the damage of waterlogging on plant growth and nitrogen uptake just after the end of waterlogging, data from experiment 1 were arranged in a split-split-plot design with years allocated as main plots, growth conditions (drained control—C and waterlogged—WL) as subplots, and waterlogging durations (T0, T7, T14, T21, T28, and T35) as sub-subplots. Four replicates were used.

To evaluate the permanent damage of waterlogging on plant growth, grain yield, grain yield components, and nitrogen uptake, data from experiment 2 were arranged in a split-plot design with years allocated as main plots and waterlogging durations as subplots. Four replicates were used.

To analyze plant growth and nitrogen uptake during recovery, data from experiments 1 and 2 were arranged, for each species, in a split-split-plot design with years allocated as main plots, growth conditions (C and WL) as subplots, and waterlogging durations (T0, T7, T14, T21, T28, and T35) as sub-subplots, with four replicates.

In all analyses, treatments were considered fixed. Significantly different means were separated at the 0.05 probability level using the Tukey test (Steel et al., 1997).

## Results

Analyses of variance for experiments 1 and 2 and for recovery revealed for both *A. sativa* and *A. byzantina* a significant interaction growth condition x waterlogging duration for most of the measured parameters. Conversely, the year mean effect and the interactions with year were not significant for all variables measured, probably because differences in temperature between the years 2015 and 2016 were small (**Figure 1**), and crops were irrigated when necessary. Accordingly, all data are presented averaged over years.

### Plant Phenology

The variety Argentina of *A. byzantina* started tillering 2–3 days earlier than the variety Genziana of *A. sativa* but reached flowering approximately 3 days later (**Table 1**). The achievement of the other growth stages differed between species by maximum 1 day.

Waterlogging did not slow plant development and the first-node-detectable stage was achieved in both species, and all treatments approximately 2 weeks after waterlogging was imposed (**Table 1**). Similarly, the plants reached flowering and maturity at the same time in both waterlogged and control conditions.

Visual observations detected chlorosis and early senescence of leaves with waterlogging durations longer than 21 days in both species, while plants remained almost disease-free during and after the treatment. 

### Experiment 1

#### Plant Growth Under Waterlogging

**Figure 2:**
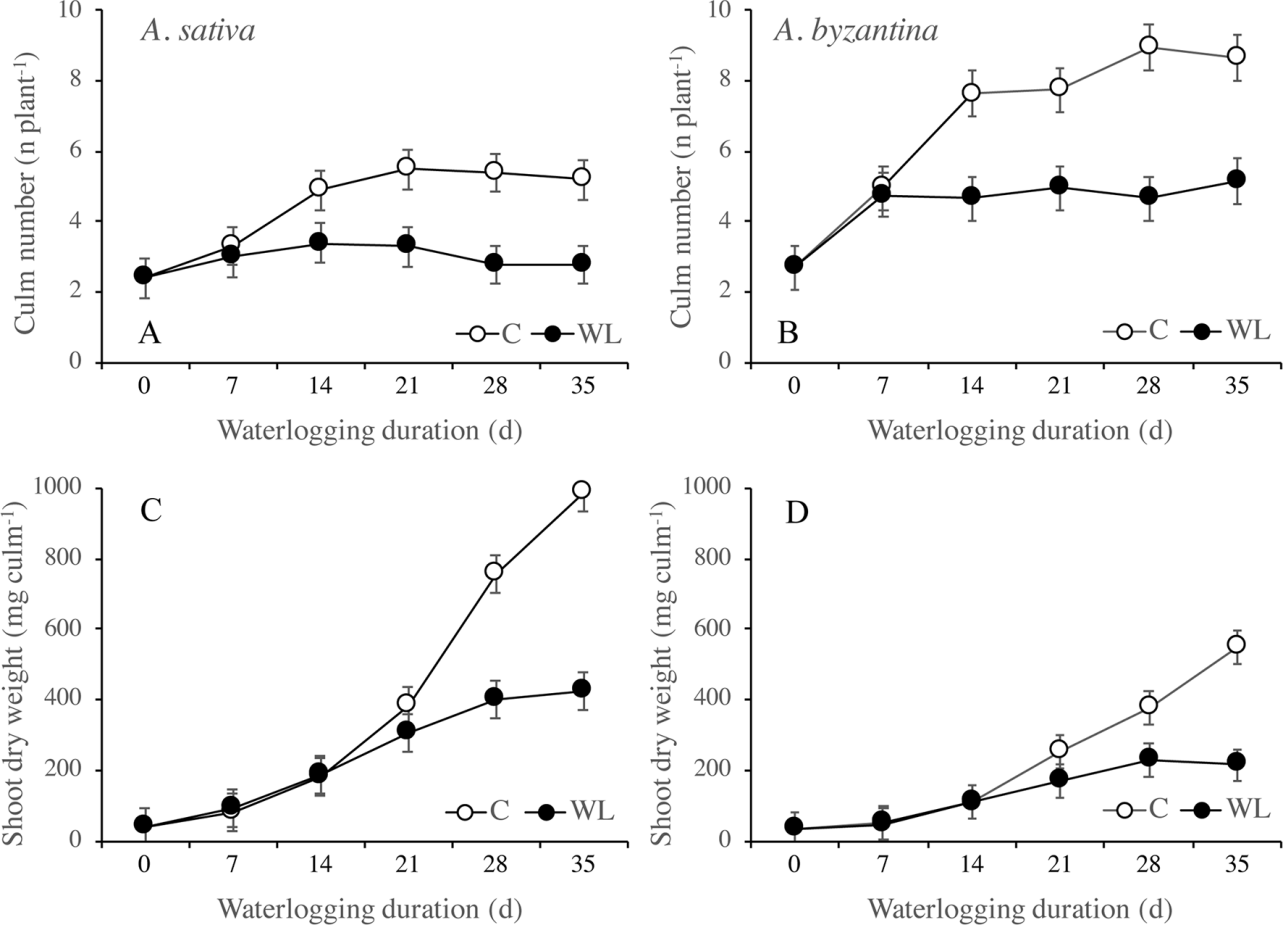
Number of culms **(A**, **B)** and shoot dry weight per culm **(C**, **D)** of *A. sativa* (left column) and *A. byzantina* (right column), as affected by the growth condition x waterlogging duration interaction. Values are means of 2 years and four replicates. Vertical bars represent HSD at P < 0.05.

**Figure 3:**
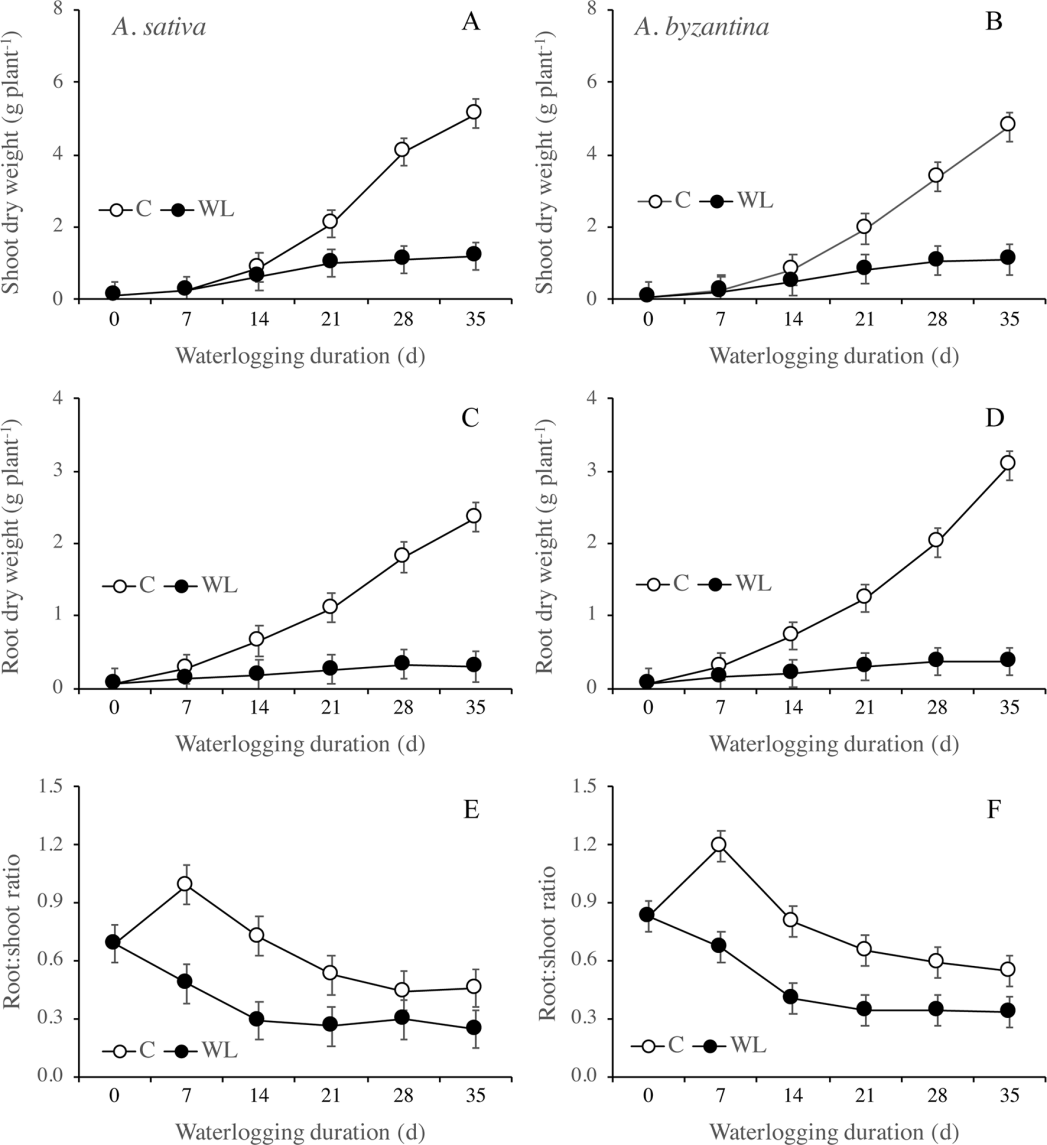
Dry weight of shoots **(A**, **B)** and roots **(C**, **D)**, and root to shoot ratio **(E**, **F)** of *A. sativa* (left column) and *A. byzantina* (right column), as affected by the growth condition x waterlogging duration interaction. Values are means of 2 years and four replicates. Vertical bars represent HSD at P < 0.05.

In the drained C plants, the number of culms per plant increased significantly between T0 and T14 but no more after then, whereas in the waterlogged plants, it increased only up to T7 (**Figures 2A, B**). At T35, control plants had approximately five culms in *A. sativa* and nine in *A. byzantina*, whereas WL plants had three and five culms, respectively. These results indicate that tillering ceased around the first-node-detectable stage in C plants and was severely inhibited under waterlogging. Treatments longer than 14 days also reduced tiller growth and stopped it after 28 days (**Figures 2C, D**). Thus, the dry matter accumulation in the shoots of both species was similar in C and WL conditions up to T14, after which it increased linearly in drained conditions and almost stopped under waterlogging (**Figures 3A, B**).

Root growth was more severely affected than shoot growth and was significantly lower in WL than in C plants from T14 on (**Figures 3C, D**). After 35 days, the dry weight of shoots of the drained plants had increased by 57 times either the species, whereas that of roots by 37 and 44 times, respectively, in *A. sativa* and *A. byzantina*. Conversely, WL plants of both species increased by only approximately 13 times in shoots and 5 times in roots. The higher and prompter sensitivity of roots to waterlogging caused the root:shoot ratio to be lower in WL than C plants (**Figures 3E, F**).

**Figure 4:**
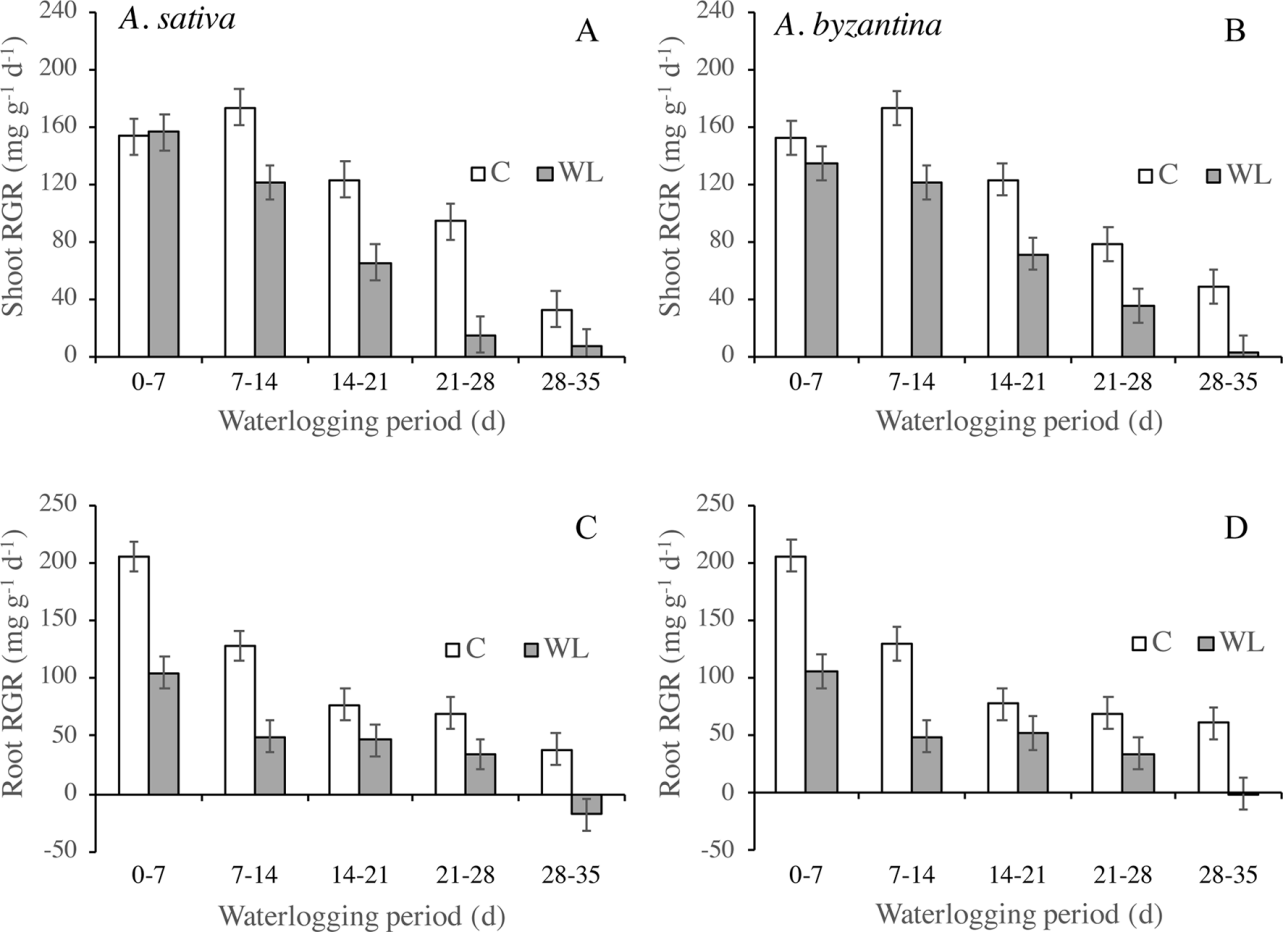
Relative growth rate (RGR) of shoots **(A**, **B)** and roots **(C**, **D)** of drained controls and waterlogged plants of *A. sativa* (left column) and *A. byzantina* (right column) during the waterlogging treatment. Values are means of 2 years and four replicates. Vertical bars represent HSD at P < 0.05.

The RGR of shoots and roots decreased progressively in both control and waterlogged plants as plant development proceeded, but values were lower in WL (**Figure 4**). In shoots, the RGR was approximately halved during the third week of waterlogging (**Figures 4A, B**), whereas in roots, this had occurred during the first week (**Figures 4C, D**). After 28 days of WL, the roots of *A. sativa* showed a negative RGR suggesting that some died roots were detached.

#### Nitrogen Uptake Under Waterlogging

The nitrogen concentration progressively decreased both in shoots and in roots of the two oats from T0 to T35, independent of the growth conditions (**Table 2**). Compared to the drained controls, WL plants showed lower N concentration in shoots. However, in *A. sativa*, the decrease was significant after 7-, 14-, and 21-day waterlogging, but not for longer exposures, while in *A. byzantina*, values differed significantly only for T14 and T21. Conversely, the N concentration of roots was never significantly affected by waterlogging. The N content of WL plants was always lower than that of controls, with differences that became significant in shoots at T14 in both species, whereas in roots at T7 in *A. sativa* and T14 in *A. byzantina* (**Table 2**).

**Table 2:** Nitrogen concentration and content of shoots and roots of *A. sativa* and *A. byzantina*, as affected by growth condition x waterlogging duration interaction.

Growth conditions	Waterlogging duration (d)	Nitrogen concentration (%)		Nitrogen content (mg plant^−1^)	
		Shoot	Roots	Shoot	Roots
		*Avena sativa*			
Control	0	3.0 ab	2.4 a	2.7 f	1.5 f
	7	3.4 a	1.7 bc	8.9 de	4.4 e
	14	2.9 ab	1.5 cde	25.9 c	9.9 d
	21	1.8 c	1.1 def	38.5 b	11.8 c
	28	1.1 de	0.8 f	46.7 a	15.1 b
	35	1.0 de	0.8 f	49.5 a	18.9 a
Waterlogged	0	3.0 ab	2.4 a	2.7 f	1.5 f
	7	2.6 b	2.1 ab	7.1 de	2.7 f
	14	1.4 cd	1.6 bcd	9.2 de	2.9 ef
	21	1.1 de	1.1 def	10.8 d	2.7 f
	28	0.6 e	1.0 ef	7.2 de	3.1 ef
	35	0.6 e	0.9 ef	6.6 e	2.5 f
		*Avena byzantina*			
Control	0	3.1 a	2.3 a	2.6 e	1.6 f
	7	3.6 a	1.6 bc	8.7 d	4.6 e
	14	3.0 a	1.2 cde	24.9 c	8.4 d
	21	2.0 b	1.0 de	38.3 b	12.5 c
	28	1.2 cd	1.0 de	41.8 b	20.7 b
	35	1.0 cd	0.7 e	47.3 a	22.7 a
Waterlogged	0	3.1 a	2.3 a	2.6 e	1.6 f
	7	2.9 a	1.9 ab	6.3 d	2.8 ef
	14	1.6 bc	1.5 bcd	8.1 d	3.1 ef
	21	1.2 cd	1.0 de	9.7 d	3.0 ef
	28	0.7 d	0.9 e	7.7 d	3.4 ef
	35	0.6 d	0.8 e	6.6 d	3.1 ef

Values are mean of 2 years and four replicates. For each species, means followed by the same letter within a column are not significantly different at P < 0.05, using the Tukey test.

**Figure 5:**
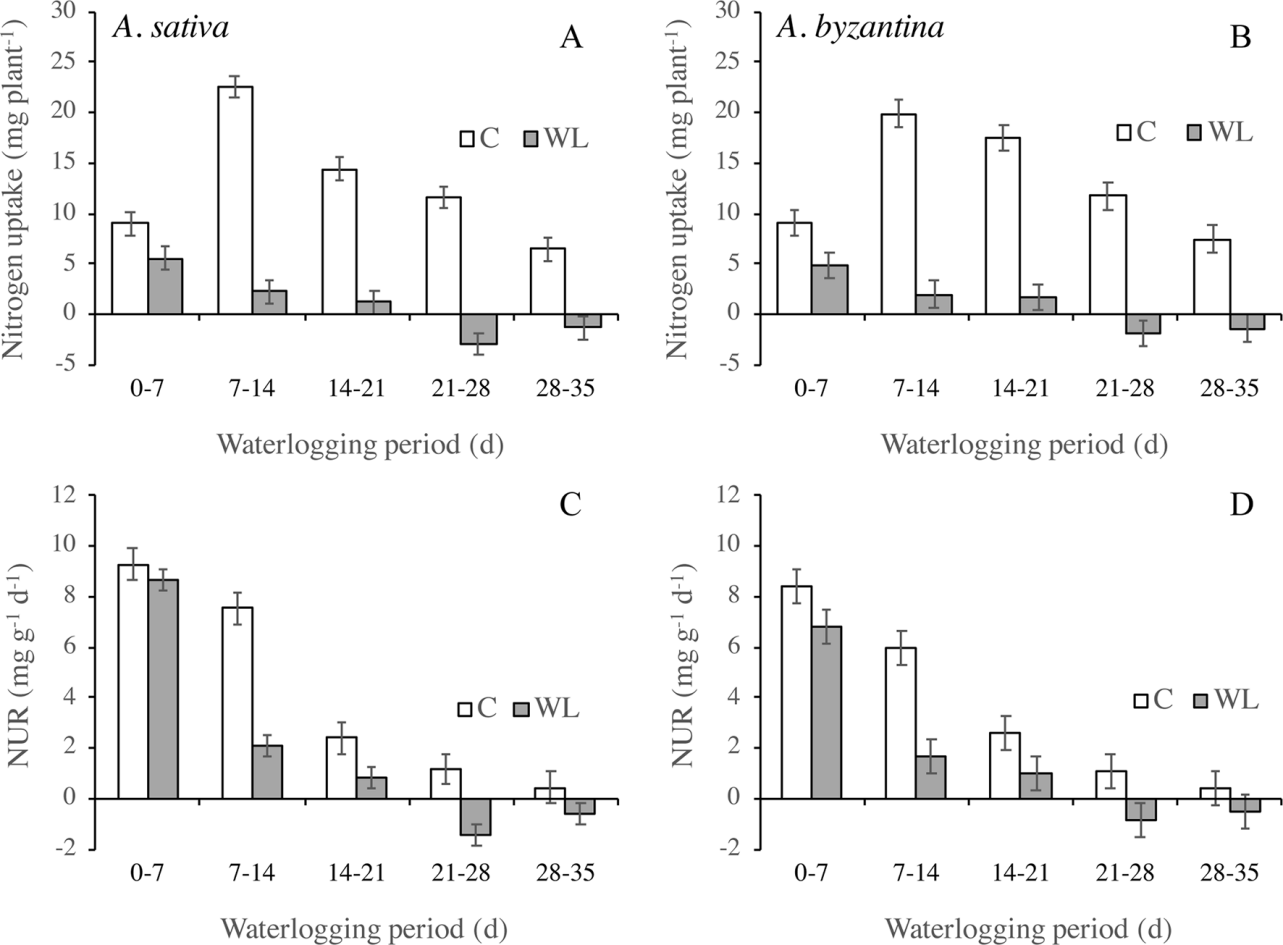
Nitrogen uptake **(A**, **B)** and net uptake rate (NUR) of nitrogen **(C**, **D)** of drained controls and waterlogged plants of *A. sativa* (left column) and *A. byzantina* (right column) during the waterlogging treatment. Values are means of 2 years and four replicates. Vertical bars represent HSD at P < 0.05.

In the two oats, the amount of N taken up by the whole plant in the first 7 days of waterlogging was approximately half that of the controls and it then decreased sharply (**Figures 5A, B**). A loss of N occurred in plants waterlogged for more than 21 days, which could be due to both N leakage from damaged root tissues or to the death and consequent detachment of roots and leaves. The amount of N taken up by plants depends on the development of the root system and the rate at which it absorbs N. The latter parameter, estimated by the NUR, decreased in both waterlogged and drained plants during the period of treatment (**Figures 5C, D**). In waterlogged plants, however, it was dramatically lower than in the controls from the second week of waterlogging on, and it became negative after 3 weeks.

### Experiment 2 and Recovery

#### Growth Rate and Nitrogen Uptake After Waterlogging

As both species reached maturity on the same date in all treatments, the recovery time decreased from 102 to 67 days with an increase of WL duration from 0 to 35 days. Thus, plants that suffered waterlogging for a shorter period had also a longer time and, therefore, better chances of recovery. To eliminate these differences in the length of time from the end of waterlogging up to maturity, the dry matter and N accumulations during recovery were expressed as daily absolute and RGR.

**Figure 6:**
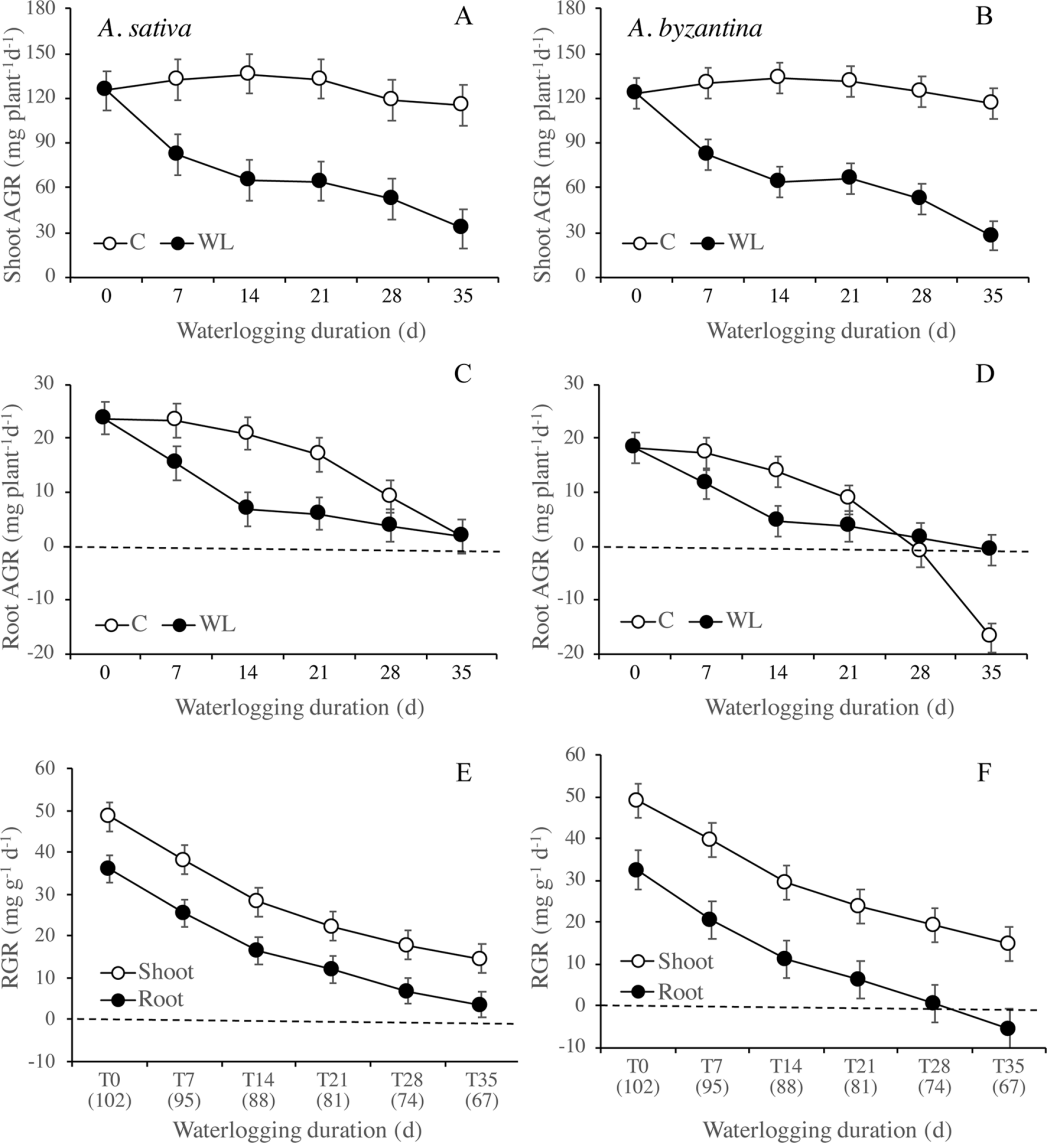
Absolute growth rate (AGR) of shoots **(A**, **B)** and roots **(C**, **D)** of *A. sativa* (left column) and *A. byzantina* (right column) during recovery, as affected by the growth condition x waterlogging duration interaction, and relative growth rates (RGR) of shoots and roots **(E**, **F)** as affected by waterlogging duration. Values of AGR are means of 2 years and four replicates. Values of RGR are means of 2 years, two growth conditions, and four replicates. Vertical bars represent HSD at P < 0.05. In brackets, the duration of recovery, in days.

Between the end of waterlogging and maturity, the AGR of the shoot was markedly lower in WL plants than in the controls, with very similar trends in the two species (**Figures 6A, B**). The AGR of control shoots was quite constant throughout the period, while in WL plants, it tended to decrease with increasing duration of exposure, demonstrating a lower AGR after longer WL. Accordingly, the difference from the controls increased from 38% (T7) to 72% in *A. sativa* and to 76% in *A. byzantina* (T35). The AGR of the roots was much lower than that of the shoot and showed a decreasing trend in both C and WL plants (**Figures 6C, D**). In controls, values decreased sharply after T21, thus suggesting that, approximately 2 weeks after the first node became detectable (T14), either root growth ceased, or it was nullified by a consistent remobilization of reserves from roots to panicles. The decrease in root growth was more pronounced in *A. byzantina*, in which root AGR became negative toward the end of the growth cycle. In the WL plants of both species, root growth was very low with all WL durations exceeding 7 days, which demonstrates that root recovery was greatly impaired after the start of stem elongation. The RGR of both shoots and roots decreased as the development proceeded, with trends that did not differ between control and waterlogged plants and were similar in the two organs (**Figures 6E, F**). No appreciable differences between species were observed in the RGR of shoots, while that of roots was always lower in *A. byzantina*.

**Figure 7:**
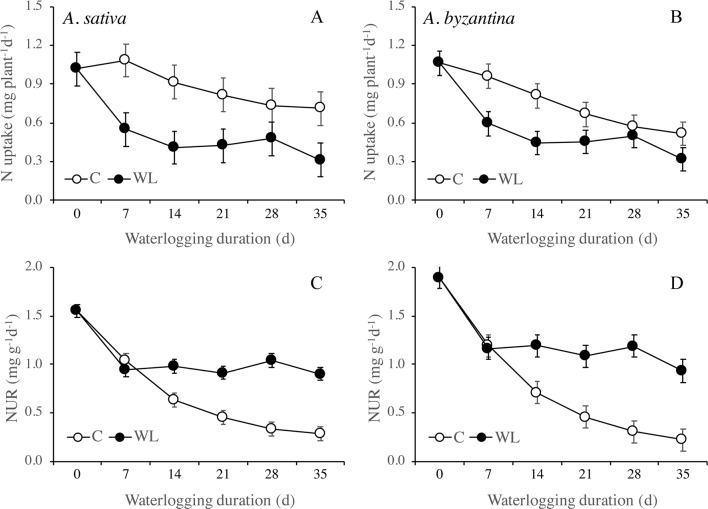
Nitrogen uptake **(A**, **B)** and net uptake rate (NUR) of nitrogen **(C**, **D)** of *A. sativa* (left column) and *A. byzantina* (right column) during recovery, as affected by the growth condition x waterlogging duration interaction. Values are means of 2 years and four replicates. Vertical bars represent HSD at P < 0.05. In brackets, the duration of recovery, in days.

Nitrogen uptake from the end of waterlogging to maturity was approximately 50% lower in previous WL plants of *A. sativa* compared to drained controls, with small differences in response to the duration of treatment (**Figure 7A**). In *A. byzantina*, the N uptake progressively decreased with the proceeding of the growth cycle in control plants, so that differences between WL and C plants were lower than in the other oat species (**Figure 7B**). The NUR for nitrogen was by 22% higher in *A. byzantina* than in *A. sativa* at T0 (**Figures 7C, D**). In the controls of both species, it showed a decreasing trend indicating that the N-uptake efficiency of roots declined with plant development. In contrast, in plants that had recovered from waterlogging, it remained relatively constant, and higher than in the controls, independently of the duration of waterlogging and/or the length of the recovery period. These results suggest that, in waterlogged oats, the smaller root systems were compensated by a higher level of uptake efficiency in the root units.

#### Waterlogging Effects on Dry Matter and Grain and Nitrogen Yields at Maturity

**Figure 8:**
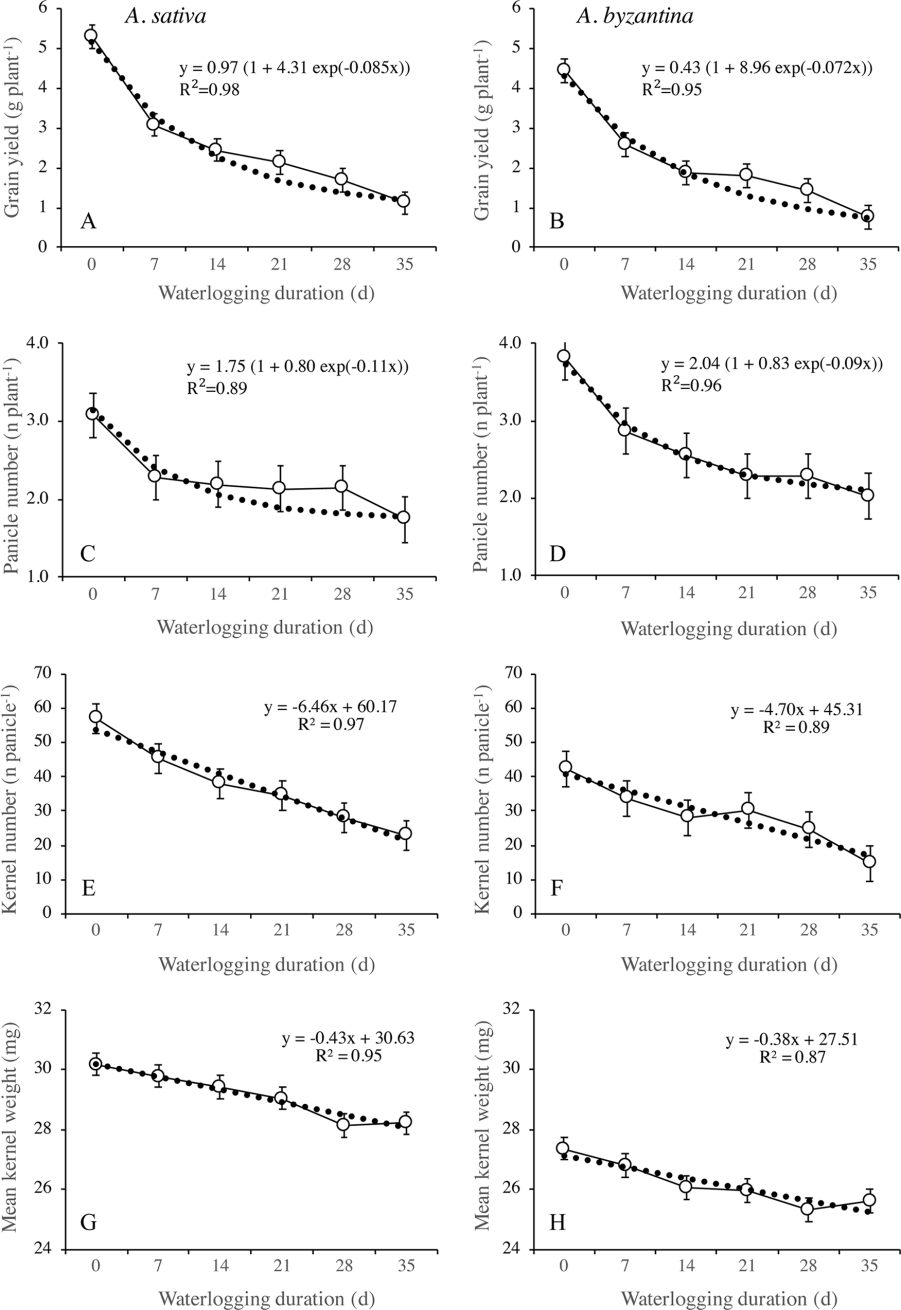
Grain yield **(A**, **B)**, number of panicles per plant **(C**, **D)**, number of kernels per panicle **(E**, **F)**, and mean kernel weight **(G**, **H)** of *A. sativa* (left column) and *A. byzantina* (right column), as affected by waterlogging duration at tillering. Values are means of 2 years and four replicates. Vertical bars represent HSD at P < 0.05.

In the drained conditions, the straw (culms+leaves+chaff) dry weight at maturity was slightly higher in *A. byzantina*, whereas that of roots was by 22% higher in *A. sativa* (**Table 3**). In addition, grain yield was by 16% higher in *A. sativa* (**Figures 8A, B**). Accordingly, *A. sativa* accounted for a higher root:shoot ratio and harvest index compared to *A. byzantina* (**Table 3**). In both species, the exposure to waterlogging at tillering significantly reduced the dry matter recorded at maturity, and straw and root biomass were approximately 35% lower after only 7 days of waterlogging in both species (**Table 3**). In straw, the decrease progressively reached 70% in *A. sativa* and 73% in *A. byzantina*, whereas in roots, it was approximately 84% in both oats. As a consequence, the straw biomass of plants waterlogged for 35 days was similar in the two species, while that of roots was by 29% higher in *A. sativa*. Patterns of decrease were similar in leaves, culms, and chaff and were associated with a significant decrease in the number of culms, which were 4.1 and 5.8 per plant in the controls of *A. sativa* and *A. byzantina*, respectively, and approximately 3 in the former and 4 in the latter in WL plants, without differences among waterlogging durations (data not shown). In both species, the root:shoot ratio was similar in controls and 7-day waterlogged plants, but markedly lower in longer treatments (**Table 3**).

**Table 3:** Vegetative biomass, root to shoot ratio, harvest index, and N content of grain, straw, and roots of *A. sativa* and *A. byzantina* at maturity, as affected by the waterlogging duration treatment at the tillering stage.

Waterlogging duration (d)	Dry matter (g plant^−1^)		Root: shoot	Harvest index (%)	Nitrogen content (mg plant^−1^)		
	Straw	Roots			Grain	Straw	Roots
	*Avena sativa*						
0	7.6 a	2.5 a	0.19 a	41.1 a	66.7 a	29.6 a	11.9 a
7	5.0 b	1.6 b	0.19 a	38.8 ab	33.3 b	19.3 b	9.4 a
14	3.9 bc	0.8 c	0.13 b	37.4 ab	26.0 c	16.8 b	5.2 b
21	4.0 bc	0.7 c	0.12 b	34.5 b	25.0 c	18.2 b	4.2 b
28	3.3 cd	0.6 c	0.12 b	33.8 b	23.0 c	19.6 b	3.1 b
35	2.2 d	0.4 c	0.12 b	33.2 b	14.3 d	12.8 c	3.0 b
	*Avena byzantina*						
0	8.2 a	1.9 a	0.15 a	35.2 a	65.7 a	32.6 a	14.2 a
7	5.4 b	1.2 b	0.16 a	31.5 ab	33.0 b	21.5 b	11.1 a
14	4.2 b	0.6 c	0.10 b	31.8 ab	26.1 c	18.2 b	5.8 b
21	4.4 b	0.6 c	0.09 b	29.1 b	24.6 c	20.1 b	4.9 b
28	3.6 bc	0.5 c	0.10 b	28.5 b	22.6 c	21.2 b	4.0 b
35	2.2 c	0.3 c	0.11 b	25.4 b	13.5 d	14.1 c	3.5 b

Values are mean of 2 years and four replicates. For each species, means followed by the same letter within a column are not significantly different at P < 0.05, using the Tukey test.

Grain yield significantly decreased with the increase of waterlogging duration, following a negative asymptotic relationship in both species (**Figures 8A, B**). Specifically, a 35-day waterlogging decreased grain yield by 79% in *A. sativa* and by 83% in *A. byzantina*, but approximately 42% of this yield was lost after only 7 days of waterlogging in both species. With the increase in waterlogging duration, the harvest index decreased progressively by 8 percent points in *A. sativa* and by 10 percent points in *A. byzantina*, highlighting that the decrease in grain yield was higher than that of straw in both species (**Table 3**).

The nitrogen concentration of all plant parts at maturity was not significantly modified by the duration of waterlogging in both species so that the differences in N content revealed those in biomass (data not reported). The N content was significantly lowered after 7-day WL duration in straw and grain, and after 14-day duration in roots, without appreciable differences between species (**Table 3**). With longer durations, the N content did not change in the roots but decreased further in straw and grain, with significant differences between T28 and T35. Overall, the plants of either *A. sativa* and *A. byzantina* subjected to the longest period of waterlogging (T35) demonstrated N content in straw, roots, and grain, of approximately 57, 75, and 79% lower than the controls, respectively.

#### Waterlogging Effects on Grain Yield Components at Maturity

**Figure 9:**
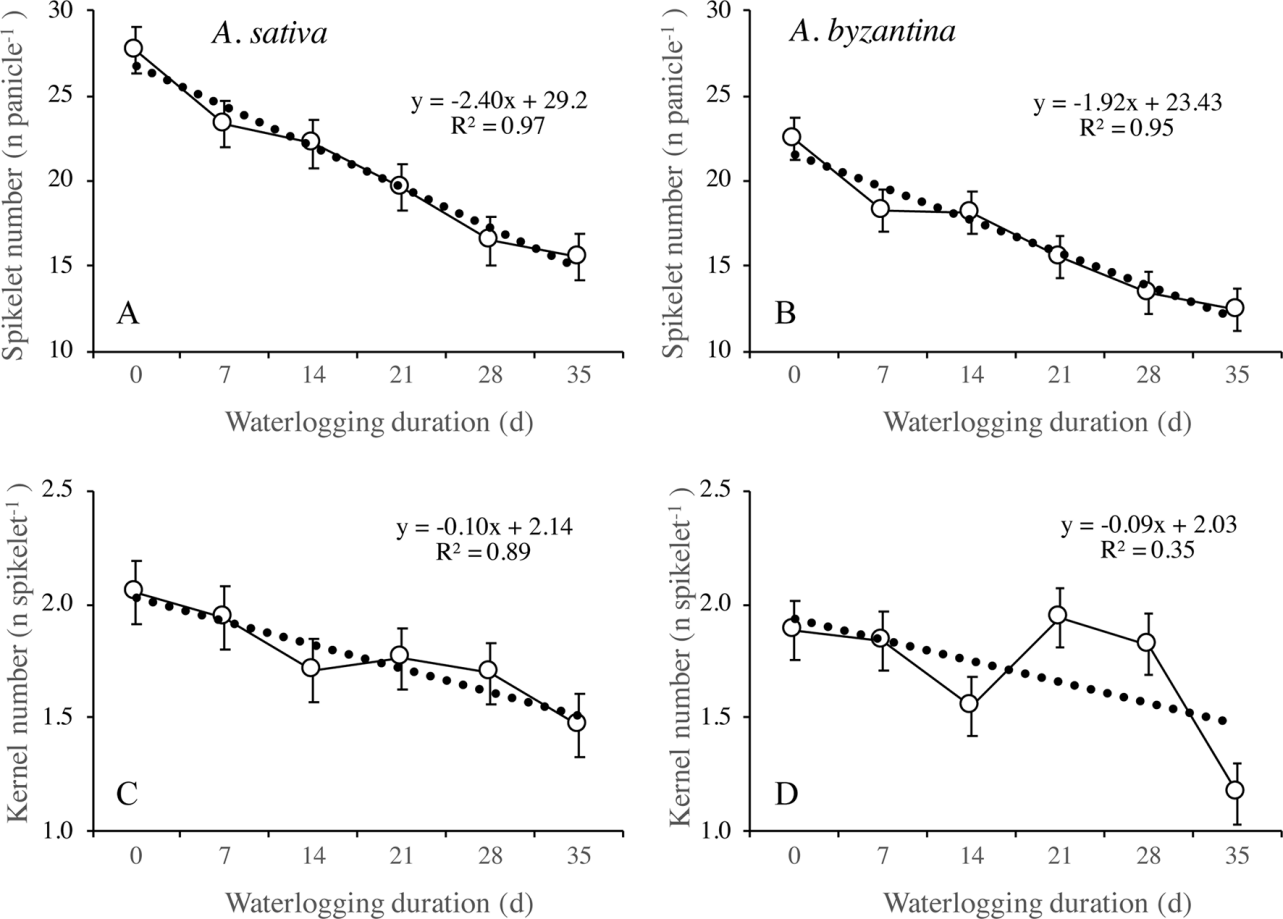
Number of spikelets per panicle **(A**, **B)** and number of kernels per spikelet **(C**, **D)** of *A. sativa* (left column) and *A. byzantina* (right column), as affected by waterlogging duration at tillering. Values are means of 2 years and four replicates. Vertical bars represent HSD at P < 0.05.

The principal components of grain yield, which are the number of panicles per plant, the number of kernels per panicle, and the mean kernel weight, were all decreased by waterlogging but differed in the rate of decrease in response to increasing WL duration (**Figures 8** and **9**). The number of panicles per plant was 3.1 and 3.8, respectively, in the controls of *A. sativa* and *A. byzantina*, and in both species, it decreased following a negative asymptotic relationship with the increase of WL duration (**Figures 8C, D**). Similar to grain yield (**Figures 8A, B**), also the number of panicles per plant decreased markedly with the 7-day WL, but then only slightly with longer durations, so that it accounted for approximately 2 and 2.3 panicles per plant in *A. sativa* and *A. byzantina* waterlogged for more than 1 week (**Figures 8C, D**). In contrast, both the number of kernels per panicle (**Figures 8E, F**) and the mean kernel weight (**Figures 8G, H**) decreased progressively with the increase of WL duration, thus following a negative linear relationship. In drained controls, the number of kernels per panicle and the mean kernel weight were both higher in *A. sativa*, by 34% the former and by 10% the latter. With the increase in WL duration, the number of kernels per panicle decreased slightly more in *A. byzantina*, so that, at T35, this parameter equaled 22.8 in *A. sativa* and 14.5 in *A. byzantina*. Conversely, the mean kernel weight decreased with similar rates in the two species.

The number of kernels per panicle can be further split into the sub-components number of spikelets per panicle and number of kernels per spikelet. In drained conditions, *A. sativa* had approximately five more spikelets per panicle (**Figures 9A, B**), whereas the number of kernels per spikelet equaled in the two species (**Figures 9C, D**). Both parameters were negatively affected by waterlogging imposed at tillering, but the patterns of decrease in response to WL duration were different: the number of spikelets per panicle followed a negative linear relationship, whereas an irregular decreasing trend was observed for the number of kernels per spikelet without appreciable differences between species.

The different patterns of decrease in response to longer WL revealed that the relative sensitivity of yield components and sub-components changed according to WL duration. With the shortest WL treatment (T7), the percentage decreases to controls ranked in the descending order: number of panicles (26%), number of kernels per panicle (21%), number of spikelets per panicle (15.7% in *A. sativa* and 18.6% in *A. byzantina*), number of kernels per spikelet (5.3% in *A. sativa* and 2.6% in *A. byzantina*), and mean kernel weight (1.3% in *A. sativa* and 2.6% in *A. byzantina*). With the longest WL treatment (T35), the decrease to controls was: kernels per panicle (60% in *A. sativa* and 62% in *A. byzantina*), panicles per plant (44% in *A. sativa* and 47% in *A. byzantina*), spikelets per panicle (44%), kernels per spikelet (29% in *A. sativa* and 32% in *A. byzantina*), and mean kernel weight (6.5%). Above figures demonstrate that waterlogging at tillering affected yield components similarly in the two oat species. However, short waterlogging decreased the number of spikelets per panicle slightly more in *A. byzantina*, which was compensated by a lower decrease in the number of kernels per spikelet. With the longest WL duration, conversely, both the number of panicles per plant and the number of spikelets per panicle were slightly more reduced in *A. byzantina*, which was responsible of the 4-percent-point higher loss in grain yield recorded in this species.

## Discussion

In this study, the physiological and agronomic tolerance of *A. sativa* and *A. byzantina* exposed to stagnant soil waterlogging at tillering was assessed by determining growth parameters and N uptake immediately after the end of waterlogging and at maturity, which occurred 2–3 months later. In order to relate the waterlogging response of oats to their specific morphological and phenological traits, our results are discussed in comparison to published results in wheat and barley, which responses to waterlogging have been investigated more deeply.

In drained conditions, *A. sativa* displayed a higher grain yield and root:shoot ratio than *A. byzantina*, but this did not influence their response to waterlogging, which similarly reduced the biomass and N uptake of the two species both during waterlogging and at maturity. Thus, in these standard European oat genotypes (Murariu et al., 2013), we did not find a diverse waterlogging tolerance, such that reported among Brazilian and Australian oat genotypes by Lemons e Silva et al. (2003) and by Setter and Waters (2003).

### Plant Growth Under Waterlogging

Plants of both species survived all of the waterlogging durations, but they suffered a strong growth reduction from exposures exceeding 7 days.

Growth was more hindered in roots than in shoots under waterlogging and ceased almost completely after 14 days in the former and after 21 days in the latter. The RGR of roots became negative after 28 days of waterlogging, which could be a consequence of the detachment of dead root fragments (Brisson et al., 2002; Ploschuk et al., 2018), which was more pronounced in *A. sativa*. Accordingly, at the end of the treatment, the WL plants had a lower root:shoot ratio compared to the drained controls and started recovery not only with a smaller shoot biomass, but also with a proportionally smaller root system supporting the growth of shoots and panicles. A higher waterlogging sensitivity of roots is generally interpreted as a direct consequence of oxygen depletion or other detrimental changes in the root environment (Herzog et al., 2016; de San Celedonio et al., 2017). However, as this different sensitivity of shoots and roots was also observed in germinating wheat seedlings that were completely submerged (Arduini et al., 2016b), we suggest that it may also be because cell division, which is much more energy demanding than cell elongation, contributes more to root growth than to shoot growth (Malik et al., 2002; Loreti et al., 2016). In our research, waterlogging reduced markedly both root and shoot RGRs in the two oats. Conversely, wheat and barley exposed to waterlogging for 14–15 days at a comparable growth stage showed only reduced root RGR, while that of shoots was similar to drained controls (de San Celedonio et al., 2017; Ploschuk et al., 2018). The detrimental effect of waterlogging on shoot growth rate observed in *A. sativa* and *A. byzantina* could be due, at least in part, to a lower ability to activate fermentative metabolism (Gibbs and Greenway, 2003). On the short period, indeed, fermentation could enable roots to withstand the energy shortage induced by anaerobiosis, thus maintaining water and mineral supply to the shoot. We found that shoot growth reduction resulted first from less tillering and second from reduced biomass accumulation per unit culm. Similar to what we hypothesized for root growing points, the inhibition of tiller production probably depended on the negative effect of anaerobiosis on the cell division of submerged tiller buds. In support, Watson et al. (1976) found that the inhibition of tiller initiation under waterlogging was more pronounced in oat than in wheat and barley.

Despite the higher growth reduction in roots compared to shoots, Malik et al. (2001) and Robertson et al. (2009) found that, in wheat, waterlogging reduced the number of adventitious roots per plant proportionally less than the number of tillers, so that the number of adventitious roots per tiller increased. According to Herzog et al. (2016), this is a strategy to overcome the reduced root efficiency caused by waterlogging. In our research, the root dry weight per culm increased from 26 to 71 and 105 mg, respectively, in *A. byzantina* and *A. sativa*, but, in contrast to the findings of Malik et al. (2001), this increment was much higher in drained controls, suggesting a lack of ability of oat roots to react to waterlogging stress. A reason of this could be that seminal roots, which are more sensitive than nodal roots, play a more prominent role during vegetative growth in oats than in wheat and barley (Bonnett, 1961; Malik et al., 2002).

### Nitrogen Uptake Under Waterlogging

Reduced shoot growth under waterlogging has been associated with lower photosynthetic CO2 assimilation rate (Pang et al., 2004), but this, however, is more likely a consequence of reduced water and mineral uptake by damaged roots, rather than a direct effect on leaf efficiency (Colmer and Greenway, 2011; de San Celedonio et al., 2017). In specific, the reduced growth of the main culm and of tillers during waterlogging was often associated with the reduced N supply to the shoot, which, in turn, leads to a lower chlorophyll content in leaves (Malik et al., 2002; Masoni et al., 2016;Arduini et al., 2016a). The insufficient nitrogen status of waterlogged plants is due to one or more of the following effects: the depletion of N in soil (Belford et al., 1985; Nguyen et al., 2018), the reduced root growth (Brisson et al., 2002; Ploschuk et al., 2018), or the “energy crisis” caused by anaerobiosis in the root environment, which impairs the active transport of nitrate against its electrochemical gradient (Gibbs and Greenway, 2003; Pang et al., 2007). Thus, it is not easy to discern whether the lower N availability to roots and/or the lower N translocation to the shoot are the cause of reduced root and shoot growth, or vice versa; it depends on the lower demand.

In both *A. sativa* and *A. byzantina*, shoot N concentration was the first parameter to be significantly reduced by waterlogging, along with root RGR. As root N concentration never differed significantly for waterlogged and control plants, while NUR was markedly decreased, we argue that waterlogging affected primary root growth and N translocation to the shoot, rather than N availability in the soil. The negative values of root RGR, plant N accumulation, and NUR recorded with the longer waterlogging durations also suggest that consistent N leakage from root tissues started after 21 days of continuous waterlogging, which was followed 1 week later by the loss of root fragments (Malik et al., 2002).

In the present research, N concentration in shoots was progressively lower than that of controls, with an increasing waterlogging duration of up to 21 days. A similar N dilution in shoot tissues has been reported in waterlogged wheat and barley, and it is probably due to the decreased N supply by the roots coupled to the maintenance of shoot growth (de San Celedonio et al., 2017; Ploschuk et al., 2018). In such a case, shoot growth can be sustained by the mobilization of nitrogen from older leaves to both younger leaves and the reproductive apex (Colmer and Greenway, 2011). According to Belford et al. (1985), the resulting lower N concentration of older leaves is primarily responsible for restricted tiller production in waterlogged wheat, and thus leaf chlorosis and reduced tillering are closely related (de San Celedonio et al., 2016; Sundgren et al., 2018). In the present research, however, reduced N concentration was associated with reduced tillering but not with leaf chlorosis, which became visible in both species only with WL treatments that completely stopped shoot growth (T21).

### Recovery From Waterlogging

From an agronomic point of view, the waterlogging tolerance of crops relies on their ability to recover at the end of the stress period, and thus achieve an acceptable yield. In winter cereals, the survival of root apices and lateral root initials under waterlogging, and the restoration of tillering upon drainage, are considered crucial plant traits to ensuring recovery (Cannell et al., 1985; Robertson et al., 2009; Hayashi et al., 2013; de San Celedonio et al., 2016; Herzog et al., 2016). The former allows plants to rapidly resume root growth to supply the shoot with adequate nutrients, and the latter is essential to replace tillers that have died or developed minor inflorescence under waterlogging.

In the present research, plants of both oat species reached maturity and produced grain with all the waterlogging durations. However, the root, straw, and grain biomass recorded at maturity were significantly lower than in the controls even with the shortest exposure, thus demonstrating that plants of *A. sativa* and *A. byzantina* were permanently damaged when they experienced a 7-day or longer waterlogging at tillering combined with spring temperatures. The current data contrast with our previous findings on barley and wheat, which showed reduced biomass and grain yield only after 16 and 20 days of waterlogging (Masoni et al., 2016; Pampana et al., 2016; Arduini et al., 2016a), and with those of Cannell et al. (1985) and Watson et al. (1976), who found that oats waterlogged at tillering recovered better than other cereals. In all these studies, waterlogging was imposed at the same growth stage as in our study, i.e., tillering, but the sowing was performed in autumn, while in the present study, it was performed in early spring conditions. Thus, we suggest that the higher temperatures during waterlogging and the shorter time to recover could, at least in part, be responsible of the higher sensitivity found in oats. The mean temperatures experienced by oats throughout the 35 days of waterlogging were, in this experiment, close to 20°C, whereas they were only approximately 6°C in our previous research into wheat (Arduini et al., 2016a). Higher temperatures increase metabolic activities and speed up plant development, so that oat plants sown in February reached the stage first-node-detectable approximately 14 days after the beginning of the waterlogging treatment (T14), whereas autumn sown barley and wheat plants reached this stage long after the end of waterlogging (Masoni et al., 2016; Arduini et al., 2016a). This may be because plants did not resume tillering during recovery, as reported by Cannell et al. (1985) and by de San Celedonio et al. (2016), and also because N application was ineffective. In support of our hypothesis, after the stage, first-node-detectable tiller production also almost ceased in the controls of both species, and the newly formed tillers did not produce panicles. Moreover, our research highlighted that despite *A. sativa* and *A. byzantina* showed different tiller production in control conditions, this trait was reduced with similar rates by waterlogging.

As root growth is generally more affected by waterlogging than shoot growth, effective recovery would involve a preferential allocation of carbon to roots to re-establish the root:shoot ratio typical of plants in drained soil (Malik et al., 2002; Robertson et al., 2009). In wheat and barley, Ploschuk et al. (2018) found that root RGR was higher in waterlogged plants than in controls just after drainage. Conversely, we found in both oats that the RGR measured over the entire recovery period equaled in WL and control plants, which induce to exclude that root growth rate was faster after waterlogging. Accordingly, the root:shoot ratio reached control values only in plants waterlogged for 7 days. In addition, through visual observations, we could not detect a higher development of crown roots in previously waterlogged plants compared to the controls. In wheat and barley seedlings, in contrast, the recovery of root mass was sustained by the vigorous initiation and elongation of nodal roots and by the proliferation of laterals on these roots (Malik et al., 2002; Pang et al., 2004). In the present research, the inability of *A. sativa* and *A. byzantina* to resume root growth after waterlogging could also depend on the advanced growth stage during recovery. Trends of AGR showed, indeed, that the allocation of resources to roots declined both in controls and waterlogged plants after the start of stem elongation, and, according to Araki et al. (2012), cereals cease to initiate new nodal roots when they approach the reproductive stage. However, while in *A. sativa*, the AGR of roots was always equal or higher in controls than in WL treatments; in *A. byzantina* control, values were markedly lower at T35, which could suggest a higher remobilization of assimilates from the roots of this species.

Poor recovery after waterlogging has often been attributed to nitrogen deficiency in soil (Robertson et al., 2009; Nguyen et al., 2018), but this was not the case in our study, as nitrogen was supplied when pots were drained. Even though the timing of N application differed according to the duration of waterlogging, the nitrogen concentration of all plant parts did not differ among treatments at maturity, suggesting that neither WL nor the time of supply affected the N status of plants during the reproductive phase. In addition, the RGR of shoots and roots were similar during recovery in previously waterlogged plants and in controls, which allows to infer that the physiological processes involved in biomass accumulation were not impaired. Thus, the lower N uptake during recovery of waterlogged plants compared to controls was more reliable, as a consequence of the smaller root system and/or the reduced request by the smaller shoots, rather than as a consequence of lower N availability in soil or lower root efficiency. The NUR for nitrogen was even higher in the waterlogged plants than in the controls, demonstrating a higher N uptake per unit root. The values were slightly higher in *A. byzantina*, which thus compensated the smaller root system with a higher uptake efficiency of roots.

The above results clearly demonstrate that the plants of *A. sativa* and *A. byzantina* did not recover from the waterlogging experienced at tillering, as they accumulated less biomass and nitrogen after the end of treatment, and consequently, at maturity, they were smaller and yielded less grain compared to the drained plants. This was essentially the consequence of the reduced growth and the damage suffered during WL exposure, as the rates of biomass accumulation and nutrient uptake proceeded similar to controls during recovery, thus suggesting that physiological processes were resumed. We argue that the more advanced growth stage at the end of waterlogging was an important reason for the scarce recovery observed in *A. sativa* and *A. byzantina* sown at the end of winter, and our hypothesis is in agreement with de San Celedonio et al. (2014), who found that delayed sowings increased the negative response of wheat to waterlogging. In addition, Peltonen-Sainio and Peltonen (1995) reported that wheat, but not oat, formed new tillers even close to anthesis in response to late N application. These findings suggest that the ability of oat plants to recover vegetative growth after waterlogging is greatly impaired when they have achieved the stem elongation phase.

### Waterlogging Impact on Grain Yield Components

Although tillering is defined as a vegetative growth phase, during this period, oats and all cereals determine the number of tillers and also the size of inflorescences (Arduini et al., 2010). Accordingly, stress conditions during tillering can strongly affect the final yield and are of crucial agronomic interest. The lower harvest index recorded in *A. sativa* and *A. byzantina* with all waterlogging durations demonstrated that grain yield was even more affected than the accumulation of vegetative biomass, which was also found in wheat (de San Celedonio et al., 2014; Pampana et al., 2016; Arduini et al., 2016a), whereas in barley, vegetative biomass and grain yield were reduced at the same rate (Masoni et al., 2016).

When wheat and barley plants were exposed to waterlogging at tillering, the most affected yield components were spike number, because of either reduced tillering (barley) or tiller fertility (wheat), and the number of kernels per spike (Amri et al., 2014; de San Celedonio et al., 2018; Sundgren et al., 2018). Due to the different architecture of wheat and barley spikes, however, the lower spike yield was related to lower spikelet fertility in the former, and to lower spikelet number in the latter (Masoni et al., 2016; Arduini et al., 2016a). Differently from in wheat and barley, oat plants exposed to WL at tillering showed higher tiller fertility, which largely compensated for the lower tiller initiation (Watson et al., 1976; Cannell et al., 1985). The lack of synchronization of the development stages of tillers with the stages of the main shoot or with each other is a specific trait of oats allowing the transition of the apical meristem of tillers from vegetative to reproductive also during main stem elongation (Bonnett, 1966). In our research, however, tiller fertility increased only in plants waterlogged for 7 and 14 days, and only by approximately 5 percent points compared to controls in both species, which we imputed to the late sowing, which caused the shortening of all growth phases, thus reducing the time for the initiation of additional panicles.

All yield components were negatively affected by waterlogging, but the relative sensitivity of those that underwent the greatest reduction, i.e., numbers of panicles per plant and kernels per panicle, varied with increasing WL duration following the former an asymptotic relationship and the latter a linear relationship.

The asymptotic trend observed in both species demonstrates that all WL durations almost equally reduced the number of panicles per plant, which could be because this component was fully established around T14, when stem elongation started. As a consequence, this trait could not recover upon drainage, as it was found by Cannell et al. (1985) in oat, and by de San Celedonio et al. (2016) in wheat and barley. Conversely, the linear decrease of the number of grains per panicle suggests that this number was not definitively fixed at the end of waterlogging, which allowed oats to resume panicle development during recovery. The number of kernels per spikelet was the panicle component that displayed the highest stability, as it achieved values close to controls even with 21- and 28-day long waterlogging. In contrast, this trait proved to be the most sensitive to waterlogging in wheat (Ghobadi et al., 2011; Marti et al., 2015; Masoni et al., 2016; Arduini et al., 2016a; de San Celedonio et al., 2018), which supports the hypothesis of Mahadevan et al. (2016) of an inverted hierarchy of plasticities in the components of grain number in wheat compared to oat. In oat, floret differentiation begins during tillering as in all cereals, but the final number of florets is established later than in wheat and barley, close to the start of grain filling. Thus, when adverse conditions during vegetative growth are followed by favorable nutrient supplies, oat plants have the capacity to compensate for the smaller panicles by filling all fertilized florets within a spikelet (Finnan and Spink, 2017). Starting from our findings and from literature, we infer that the N supply to plants waterlogged at tillering differently affects the yield components of winter cereals; in that, it sustains floret differentiation in oats, whereas it promotes tillering in wheat (Watson et al., 1976; Peltonen-Sainio and Peltonen, 1995; Robertson et al., 2009). Grain yield per plant was slightly higher in *A. sativa* than in *A. byzantina*, primarily due to the higher number of spikelets per panicle and mean kernel weight. Despite these differences, patterns of decrease in response to waterlogging followed similar trends, and after 7-day waterlogging, they both lost 42% of grain yield. The further decrease of grain yield with increasing WL durations was slightly higher in *A. byzantina* causing a 4-percent-point higher yield loss, which, however, we do not consider to be enough to suggest a higher tolerance to waterlogging of *A. sativa*.

Mean kernel weight is the last determined yield component in cereals, and it was not affected by waterlogging at tillering in oat, wheat, and barley (Cannell et al., 1985; Robertson et al., 2009; Masoni et al., 2016; Pampana et al., 2016), probably because plants adjusted kernel size to compensate for the lower number. In contrast, this parameter was found to be sensitive to waterlogging imposed later in the growth cycle, primarily because of reduced ovary growth (Watson et al., 1976; Araki et al., 2012; de San Celedonio et al., 2014; Marti et al., 2015). In our research, the slight decrease in mean kernel weight recorded in both oat species was probably due to reduced grain filling, driven by either the lower assimilation during recovery or the smaller amount of pre-anthesis resources to be remobilized (Li et al., 2011).

## Conclusion

*A. sativa* and *A. byzantina* sown at the end of winter and exposed to stagnant waterlogging for 7 to 35 days from the two-tiller stage onwards, showed markedly reduced grain yield starting from the shortest exposure, primary due to the severe damages suffered under waterlogging.

In both species, the first parameters that decreased under waterlogging were the RGR of roots and the N concentration of shoots, followed by the number of tillers per plant, which suggests that root growing points and tiller buds were the first targets of waterlogging stress. During recovery, the RGR and the N NUR either achieved control values or were even higher, highlighting that the physiological processes involved in dry matter accumulation and mineral uptake were resumed upon drainage. In contrast, damage to growing points appeared to be permanent and plants were unable to initiate new roots and new tillers, probably because of their advanced growth stage during recovery.

The sensitivity of grain yield components changed according to waterlogging duration, so that grain yield loss was primarily due to reduced tiller and panicle initiation with short waterlogging, whereas to reduced panicle number and also panicle size with longer durations. These results demonstrate that oat plants partially compensated the lower number of tillers with higher tiller and spikelet fertilities after short waterlogging. This is to impute to the asynchronous and longer phase of panicle differentiation and to the determination of the number of kernels per panicle close to grain filling, which is a specific trait of oat compared to other winter cereals that produce spikes, such as wheat and barley.

In the present research, we did not screen for waterlogging tolerance among *A. sativa* and *A. byzantina* genotypes, but we chose standard cultivars that are widely used in our environment because of stabile yields, which probably also includes the ability to cope with occasional soil waterlogging. The two species responded with similar patterns to increasing waterlogging duration. Thus, our results demonstrated that late sown oats were not able to produce acceptable yield, primarily because they did not resume tillering after the end of waterlogging, and the plasticity of panicle components was not able to compensate for the reduced panicle number.

This study contributes to the understanding of oat response to waterlogging through the detailed analysis of the morphological traits and the nitrogen uptake and distribution patterns which are more or less affected under waterlogging and are more or less able to recover after the stress. The comparison with published results in wheat and barley allows to highlight oat-specific traits in response to waterlogging.

## Data Availability

The raw data supporting the conclusions of this manuscript will be made available by the authors, without undue reservation, to any qualified researcher.

## Author Contributions

All authors contributed equally in planning and conducting the experiment, so as in the elaboration of data and in the preparation of the manuscript.

## Conflict of Interest Statement

The authors declare that the research was conducted in the absence of any commercial or financial relationships that could be construed as a potential conflict of interest.

## References

[B1] Amri M. El Ouni M. H. Salem M. B. (2014). Waterlogging affect the development, yield and components, chlorophyll content and chlorophyll fluorescence of six bread wheat genotypes (*Triticum aestivum* L.). Bulg. J. Agric. Sci. 20 (3), 647–657.

[B2] AOAC, Association of Official Analytical Chemists (2005). Official methods of analysis. 17th ed Gaithersburg, MD: AOAC International.

[B3] Araki H. Hossain M. A. Takahashi T. (2012). Waterlogging and hypoxia have permanent effects on wheat root growth and respiration. J. Agron. Crop Sci. 198, 264–275. 10.1111/j.1439-037X.2012.00510.x

[B4] Arduini I. Ercoli L. Mariotti M. Masoni A. (2010). Coordination between plant and apex development in Hordeum vulgare spp. distichum. C. R. Biol. 333, 454–460. 10.1016/j.crvi.2010.01.003 20451887

[B5] Arduini I. Orlandi C. Pampana S. Masoni A. (2016a). Waterlogging at tillering affects spike and spikelet formation in wheat. Crop Pasture Sci. 67, 703–711. 10.1071/CP15417

[B6] Arduini I. Orlandi C. Ercoli L. Masoni A. (2016b). Submergence sensitivity of durum wheat, bread wheat and barley at the germination stage. Ital. J. Agron. 11 (706), 100–106. 10.4081/ija.2016.706

[B7] Belford R. K. Cannell R. Q. Thomson R. J. (1985). Effects of single and multiple waterloggings on the growth and yield of winter wheat on a clay soil. J. Sci. Food Agric. 36, 142–156. 10.5539/ijb.v1n2p87

[B8] Bonnett O. T. (1961). The oat plant: its histology and development. Bulletin 672. Urbana, IL: University of Illinois Agricultural Experiment Station, 7–112. Available at: http://hdl.handle.net/2142/8676.

[B9] Bonnett O. T. (1966). Inflorescences of maize, wheat, rye, barley, and oats: their initiation and development. Bulletin 721. Urbana, IL: University of Illinois Agricultural Experiment Station, 92–102. Available at: http://www.ideals.illinois.edu/bitstream/handle/2142/27945/inflorescencesof721bonn.pdf?sequence=1

[B10] Brisson N. Rebière B. Zimmer D. Renault P. (2002). Response of the root system of a winter wheat crop to waterlogging. Plant Soil 243, 43–55. 10.1023/A:1019947903041

[B11] Bronson K. F. Fillery I. R. P. (1998). Fate on nitrogen-15-labelled urea applied to wheat on a waterlogged texture-contrast soil. Nutr. Cycl. Agroecosys. 51, 175–183. 10.1023/A:1009725900571

[B12] Browne R. A. White E. M. Burke J. I. (2006). Responses of developmental yield formation processes in oats to variety, nitrogen, seed rate and plant growth regulator and their relationship to quality. J. Agric. Sci. 144, 533–545. 10.1017/S0021859606006538

[B13] Cannell R. Q. Belford R. K. Blackwell P. S. Govi G. Thomson R. J. (1985). Effects of waterlogging on soil aeration and on root and shoot growth and yield of winter oats (*Avena sativa* L.). Plant Soil 85, 361–373. 10.1007/BF02220191

[B14] Colmer T. D. Greenway H. (2011). Ion transport in seminal and adventitious roots of cereals during O_2_. deficiency. J. Exp. Bot. 62, 39–57. 10.1093/jxb/erq271 20847100

[B15] de San Celedonio R. P. Abeledo L. G. Miralles D. J. (2014). Identifying the critical period for waterlogging on yield and its components in wheat and barley. Plant Soil 378, 265–277. 10.1007/s11104-014-2028-6

[B16] de San Celedonio R. P. Abeledo L. G. Brihet J. M. Miralles D. J. (2016). Waterlogging affects leaf and tillering dynamics in wheat and barley. J. Agron. Crop Sci. 202, 409–420. 10.1111/jac.12151

[B17] de San Celedonio R. P. Abeledo L. G. Mantese A. I. Miralles D. J. (2017). Differential root and shoot biomass recovery in wheat and barley with transient waterlogging during preflowering. Plant Soil 417, 481–498. 10.1007/s11104-017-3274-1

[B18] de San Celedonio R. P. Abeledo L. G. Miralles D. J. (2018). Physiological traits associated with reductions in grain number in wheat and barley under waterlogging. Plant Soil 429, 469–481. 10.1007/s11104-018-3708-4

[B19] Engels C. (1993). Differences between maize and wheat in growth-related nutrient demand and uptake of potassium and phosphorus at suboptimal root zone temperatures. Plant Soil 150, 129–138. 10.1007/BF00779183

[B20] Finnan J. M. Spink J. (2017). Identification of yield limiting phenological phases of oats to improve crop management. J. Agric. Sci. 155, 1–17. 10.1017/S0021859616000071

[B21] Ghobadi M. E. Ghobadi M. (2010). Effect of anoxia on root growth and grain yield of wheat cultivars. World Acad. Sci. Eng. Technol. 70, 85–88.

[B22] Ghobadi M. E. Ghobadi M. Zebarjadi A. (2011). The response of winter wheat to flooding. World Acad. Sci. Eng.Technol. 78, 440–442.

[B23] Gibbs J. Greenway H. (2003). Mechanisms of anoxia tolerance in plants. I. Growth, survival and anaerobic catabolism. Funct. Plant Biol. 30, 1–47. 10.1071/PP98095 32688990

[B24] Hayashi T. Yoshida T. Fujii K. Mitsuya S. Tsuji T. Okada Y. (2013). Maintained root length density contributes to the waterlogging tolerance in common wheat (*Triticum aestivum* L.). Field Crops Res. 152, 27–35. 10.1016/j.fcr.2013.03.020

[B25] Herzog M. Striker G. G. Colmer T. D. Pedersen O. (2016). Mechanisms of waterlogging tolerance in wheat–a review of root and shoot physiology. Plant Cell Environ. 39, 1068–1086. 10.1111/pce.12676 26565998

[B26] Hunt R. (1990). Basic growth analysis: plant growth analysis for beginners. London: Unwin Hyman. 10.1007/978-94-010-9117-6

[B27] Lemons e Silva C. F. de Mattos L. A. T. de Oliveira A. C. de Carvalho F. I. F. de Freitas F. A. dos Anjos e Silva S. D. (2003). Flooding tolerance in oats. J. New Seeds 54, 29–42. 10.1300/J153v05n04_03

[B28] Li C. Dong J. Wollenweber B. Li Y. Dai T. Cao W. (2011). Waterlogging pretreatment during vegetative growth improves tolerance to waterlogging after anthesis in wheat. Plant Sci. 180, 672–678. 10.1016/j.plantsci.2011.01.009 21421417

[B29] Loreti E. van Veen H. Perata P. (2016). Plant responses to flooding stress. Curr. Opin. Plant Biol. 33, 64–71. 10.1016/j.pbi.2016.06.005 27322538

[B30] Mahadevan M. Calderini D. F. Zwer P. K. Sadras V. O. (2016). The critical period for yield determination in oat (*Avena sativa* L.). Field Crops Res. 199, 109–116. 10.1016/j.fcr.2016.09.021

[B31] Malik A. I. Colmer T. D. Lambers H. Schortemeyer M. (2001). Changes in physiological and morphological traits of roots and shoots of wheat in response to different depths of waterlogging. Aust. J. Plant Physiol. 28, 1121–1131. 10.1071/PP01089

[B32] Malik A. I. Colmer T. D. Lambers H. Setter T. L. Schortemeyer M. (2002). Short-term waterlogging has long-term effects on the growth and physiology of wheat. New Phytol. 153, 225–236. 10.1046/j.0028-646X.2001.00318.x

[B33] Marti J. Savin R. Slafer G. A. (2015). Wheat yield as affected by length of exposure to waterlogging during stem elongation. J. Agron. Crop Sci. 201, 473–486. 10.1111/jac.12118

[B34] Masoni A. Pampana S. Arduini I. (2016). Barley response to waterlogging duration at tillering. Crop Sci. 56, 2722–2730. 10.2135/cropsci2016.02.0106

[B35] Meier U. (2001). Growth stages of mono and dicotyledonous plants. BBCH. Monograph. Berlin and Braunschweig: Federal Biological Research Centre for Agriculture and Forestry.

[B36] Murariu D. Placinta D. D. Germeier C. U. Annamaa K. Antonomova N. Bulinska-Radomska Z. (2013). Quality characteristics of European Avena genetic resources collections. Rom. Agric. Res. 30, 45–55.

[B37] Mustroph A. (2018). Improving flooding tolerance of crop plants. Agronomy 8, 160. 10.3390/agronomy8090160

[B38] Nguyen L. T. T. Osanai Y. Anderson I. C. Bange M. P. Braunack M. Tissue D. T. (2018). Impacts of waterlogging on soil nitrification and ammonia-oxidizing communities in farming system. Plant Soil 426, 299–311. 10.1007/s11104-018-3584-y

[B39] Pampana S. Masoni A. Arduini I. (2016). Grain yield of durum wheat as affected by waterlogging at tillering. Cereal Res. Commun. 44, 706–716. 10.1556/0806.44.2016.026

[B40] Pang J. Zhou M. Mendham N. Shabala S. (2004). Growth and physiological responses of six barley genotypes to waterlogging and subsequent recovery. Aust. J. Agr. Res. 55, 895–906. 10.1071/AR03097

[B41] Pang J. Ross J. Zhou M. Mendham N. Shabala S. (2007). Amelioration of detrimental effects of waterlogging by foliar nutrient sprays in barley. Funct. Plant Biol. 34, 221–227. 10.1071/FP06158 32689348

[B42] Peltonen-Sainio P. Peltonen J. (1995). Floret set and abortion in oat and wheat under high and low nitrogen regimes. Eur. J. Agron. 4, 253–262. 10.1016/S1161-0301(14)80052-X

[B43] Ploschuk R. A. Miralles. D. J. Colmer T. D. Ploschuk E. L. Striker G. G. (2018). Waterlogging of winter crops at early and late stages: impacts on leaf physiology, growth and yield. Front. Plant Sci. 9, 1863. 10.3389/fpls.2018.01863 30619425 PMC6306497

[B44] Rasaei A. Ghobadi M. E. Jalali-Honarmand S. Ghobadi M. Saeidi M. (2012). Impacts of waterlogging on shoot apex development and recovery effects of nitrogen on grain yield of wheat. Eur. J. Exp Biol. 2 (4), 1000–1007. www.pelagiaresearchlibrary.com.

[B45] Robertson D. Zhang H. Palta J. A. Colmer T. Turner N. C. (2009). Waterlogging affects the growth, development of tillers, and yield of wheat through a severe, but transient, N deficiency. Crop Pasture Sci. 60, 578–586. 10.1071/CP08440

[B46] Sasidharan R. Bailey-Serres J. Ashikari M. Atwell B. J. Colmer T. D. Fagerstedt K. (2017). Community recommendations on terminology and procedures used in flooding and low oxygen stress research. New Phytol. 214, 1403–1407. 10.1111/nph.14519 28277605

[B47] Setter T. L. Waters I. (2003). Review of prospects for germ plasm improvement for waterlogging tolerance in wheat, barley and oats. Plant Soil 253, 1–34. 10.1023/A:1024573305997

[B48] Sonego M. Moot D. J. Jamieson P. D. Martin R. J. Scott W. R. (2000). Apical development in oats predicted by the leaf stage. Field Crops Res. 65, 79–86. 10.1016/S0378-4290(99)00073-8

[B49] Steel R. G. D. Torrie J. H. Dickey D. A. (1997). Principles and procedure of statistics: a biometrical approach. New York: McGraw-Hill.

[B50] Sundgren T. K. Uhlen A. K. Waalen W. Lillemo M. (2018). Field screening of waterlogging tolerance in spring wheat and spring barley. Agronomy 8, 38. 10.3390/agronomy8040038

[B51] Watson E. R. Lapins P. Barron R. J. W. (1976). Effect of waterlogging on the growth, grain and straw yield of wheat, barley and oats. Aus. J. Exp. Agr. 16, 114–122. 10.1071/EA9760114

